# Determination of Bupropion and Its Impurities via a Chaotropic Chromatography Method Following Analytical Quality-by-Design Principles for Method Development

**DOI:** 10.3390/ph15101196

**Published:** 2022-09-28

**Authors:** Kostas Gkountanas, Anđelija Malenović, Yannis Dotsikas

**Affiliations:** 1 Laboratory of Pharmaceutical Analysis, Faculty of Pharmacy, National and Kapodistrian University of Athens, Panepistimioupoli Zografou, GR-157 71 Athens, Greece; 2 Greek Military Pharmaceutical Laboratories, Pireos 174, Tavros, GR-117 78 Athens, Greece; 3 Department of Drug Analysis, Faculty of Pharmacy, University of Belgrade, Vojvode Stepe 450, 11152 Belgrade, Serbia

**Keywords:** bupropion, chaotropic chromatography, experimental design, impurities, quality-by-design

## Abstract

A novel chaotropic chromatography method for the quantitative determination of bupropion and its impurities, following analytical quality-by-design (AQbD) principles, is presented. The analytical target profile (ATP) was defined on the basis of the efficient separation and reliable determination of bupropion and its five impurities in tablets. Preliminary experiments revealed the need for the addition of a gradient elution part. A screening fractional factorial experimental design was employed to select the critical method parameters (CMPs) and a Box–Behnken design (BBD) was utilized to investigate their influence on predefined critical method attributes (CMAs). In order to compute the design space (DS), where CMPs meet predefined acceptance limits with a high level of probability (*π* ≥ 85%), Monte Carlo simulations were performed. The working point selected from the DS corresponded to the following conditions: 37.5% acetonitrile at the start of the gradient program (up to 70% at the end of the gradient program), 45 mM of potassium hexafluorophosphate in the water phase, and the start of the linear gradient step in the gradient program at 10 min. The method was validated according to ICH guidelines and applied to the analysis of Wellbutrin^®^ tablets containing bupropion hydrochloride.

## 1. Introduction

Bupropion hydrochloride (BUP) or amfetabutamone ((±)-2-tert-butyl-amino)-3-chloropriophenone hydrochloride) is an aminoketone, administrated as a second-generation antidepressant. It behaves as a selective inhibitor of neuronal reuptake of catecholamines, noradrenaline, and dopamine [1]. It is also used in the management of smoking cessation, acting as a nicotine receptor antagonist by inhibiting nicotinic acetylcholine receptors [2].

Several HPLC-UV [3,4,5,6] and HPLC-MS [7,8,9,10] methods have been reported for the determination of BUP and its major metabolites in biological fluids. Furthermore, the stability of BUP has been studied revealing four degradation impurities in alkaline pH [11], whereas three new impurities have been identified based on an LC-MS method [12]. A literature survey revealed a single HPLC method for the separation and determination of one related impurity and two alkaline degradants [13]. Finally, the official monographs of bupropion hydrochloride extended-release tablets and bupropion hydrochloride raw material in USP Pharmacopeia list three and two related substances of BUP, respectively [14]. To the best of our knowledge, there is no other report of analytical method for the simultaneous analysis of BUP and its related impurities.

Chaotropic chromatography refers to the addition of ion interaction agents referred to as chaotropic agents in the mobile phase of RP-HPLC systems. These agents act by reducing the hydrophobic effects through ion–macromolecule interactions, or by disrupting the network of hydrogen bonds between water molecules in aqueous solutions. Anions that are used in chaotropic chromatography can be classed according to increasing chaotropic effect, which is parallel with the ability to increase basic analytes’ retention:

H_2_PO_4_^−^ < HCOO^−^ < CH_3_SO_3_^−^ < Cl^−^ < NO_3_^−^ < CF_3_COO^−^ < BF_4_^−^ < ClO_4_^−^ < PF_6_^−^

Chaotropic chromatography offers significant advantages in the adjustment of the retention and in the improvement in peak symmetry and separation efficiency of basic analytes [15,16,17]. Recent studies enabled a better understanding of fundamental and practical issues of this type of chromatography, such as the role of pH value of the aqueous phase or the concentration of chaotropic salt on the retention behavior [18,19,20].

Quality by design (QbD) constitutes a major topic in pharmaceutical development incorporating prior scientific knowledge, implementation of experimental design, and risk management during the lifecycle of a product [21]. Analytical method development remains a crucial part of pharmaceutical development and utilizes the QbD methodology to a great extent. In particular, the implementation of the analytical QbD (AQbD) methodology in liquid chromatography method development for the determination of active pharmaceutical ingredients (APIs) and their impurities has been extensively reported [22,23,24,25]. Developing an analytical method based on AQbD methodology is considered as state-of-the-art, avoiding time and money-wasting optimization approaches. The outcome is the development of a robust and reliable analytical method as AQbD provides assurance on the quality of data obtained by this method. Modern statistic tools are utilized, such as design of experiments (DoΕ) [26], which achieves a more precise and efficient optimization through the establishment of design space (DS) [27]. The computation of DS by the DoE methodology and risk assessment to evaluate likelihood of the method to produce acceptable results is also known as robust optimization.

**Figure 1:**
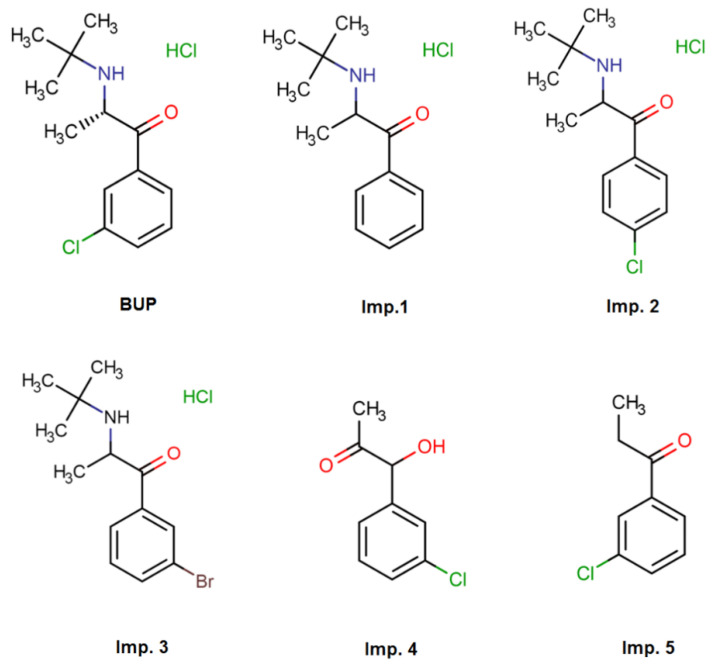
Chemical structures of BUP and its 5 impurities.

The aim of this study was to develop and optimize a selective and robust HPLC method for determining BUP and its five impurities (Figure 1) in tablets, using a chaotropic salt as a mobile-phase additive, based on the principles of AQbD. Finally, the developed method was validated according to ICH guidelines in order to prove its suitability for the intended purpose.

## 2. Results and Discussion

### 2.1. Preliminary Experiments and Screening Design

From the initial experiments, it was clear that the separation of BUP from impurity 2 and impurity 3 would be very demanding. Taking into account that these three analytes are hydrochloric salts of basic compounds and that chaotropic chromatography is suitable for such analyses, i.e., by reducing peak width and affecting their retention, we soon moved to chaotropic chromatography. Potassium hexafluorophosphate was chosen as an agent with very strong chaotropic agent and a series of preliminary experiments took place.

Following the AQbD principles in this study, firstly, we had to define the analytical target profile (ATP), which is considered as the basis of the design, and the critical method attributes (CMAs). Regarding ATP in this study, the objective was to develop an HPLC method for the reliable quantitation of BUP and its five impurities in the presence of excipients. Considering the CMAs, the criteria that had to be estimated to control the method’s performance defined in the ATP were clarified after conducting some preliminary experiments.

Concerning BUP imp. 2 and imp. 3, the similarity both in their structure and their logD values had preluded the difficulty of achieving an efficient baseline separation among these three analytes. Furthermore, BUP, imp. 1–4 have basic centers in their molecules, except for imp. 5 which is neutral. Its neutral character keeps this analyte mainly unaffected from the presence of chaotropic salt in the mobile phase and its pH adjustment. These two pieces of evidence set out the choice of gradient elution and determined the CMAs. The additional important remark was on the most polar analyte, imp. 1, which was eluting the first. Its retention behavior implicated the isocratic step preceding the linear gradient step in the gradient program. The selected CMAs were the separation between BUP and imp. 2; the separation between imp. 2 and imp. 3 and the retention factor of imp. 5, which was the last eluting peak.

The next step was the selection of CMPs, the factors that strongly determine the CMAs. These had to be wisely chosen, considering the complexity of developing an HPLC method and the great number of factors involved in this procedure. Preliminary experiments revealed the CMPs, forming the factors of a screening design. The latter had to be performed in order to reduce the number of the factors and keep those that had statistically significant effected the predefined responses.

For this purpose, a 2^6-2^ fractional factorial design was created, so as to identify these factors and investigate the knowledge space. Six quantitative factors were chosen: (A) the pH of the aqueous part of the mobile phase (2.4–4.0); (B) the ratio of acetonitrile at the start of the linear gradient step (37–43%); (C) the concentration of the chaotropic agent (20–80 mM); (D) the column temperature (30–40 °C); (E) the linear gradient step start time (5–8 min; and (F) the linear gradient end time (9–13 min). The separation criterion (s = t_2b_ − t_1e_) between adjacent peaks, where t_1e_ represents the retention time of the end of the first peak and t_2b_ the retention time of the beginning of the second peak, as well as the retention time of the last peak, was considered. Finally, the determined design responses were: t_e_bup_, t_b_imp2_, t_e_imp2_, t_b_imp3,_ and t_R_imp5_. Table 1 presents the factors and the obtained responses of the screening design, consisting of 19 experiments, as proposed by the software.

The statistical significance of factor effects on the responses was recognized through half-normal probability plots and Pareto charts. According to these graphs, (i) the ratio of acetonitrile at the start of the linear gradient step and (ii) the concentration of the chaotropic agent were found to have a statistically significant effect on the responses t_e_bup_, t_b_imp2_, t_e_imp2,_ and t_b_imp3_, whereas the same applies for (i) the gradient end time and (ii) the ratio of acetonitrile at the start of the linear gradient step for t_R_imp5_. As was anticipated, the neutral character of imp. 5 rendered its chromatographic behavior unaffected by the presence of the chaotropic salt.

### 2.2. Robust Optimization of the Chromatographic Conditions Based on AQbD

The next phase included the robust optimization step of the aforementioned significant factors, so as to establish the method’s design space, employing a typical tool of response surface methodology (RSM), the BBD. The factor gradient end time was replaced by the linear gradient step start time in order to ascertain the absence of any baseline drift during the elution of BUP, imp. 2, and imp. 3, whereas a time limit of 17 min was set to the gradient elution, so as to reduce t_R_imp5_. At this point, it should be stated that the obtained values of t_R_imp5,_ with the aforementioned limit, were acceptable despite the changes in the factors’ values; therefore, this response was not determined further. The pH was kept at 2.5 by using hydrochloric acid, ensuring the complete protonation of the basic analytes, and column temperature at 35 °C, as they were not estimated to have a significant effect during the preliminary study. The limits of the significant factors were modified at an extension, based on our experience and the experimentally obtained values from the screening design. Hence, three CMPs, (A) the ratio of acetonitrile at the start of the linear gradient step (37–41%), (B) the concentration of chaotropic agent (30–60 mM), and (C) the linear gradient step start time (7–11 min) were selected for robust optimization. Regarding the CMAs, t_e_bup_, t_b_imp2_, t_e_imp2,_ and t_b_imp3_ were clarified for this set of 16 experiments, including three central point runs, as proposed from the software for the BBD (Table 2).

**Figure 2:**
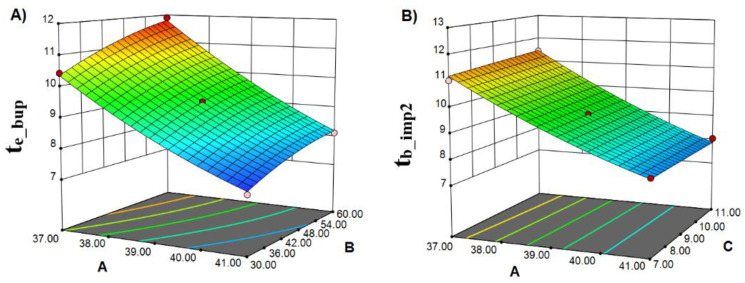
Three-dimensional graphs of (**A**) t_e_bup_ = *f* (%ACN at the start of the gradient, salt concentration—mM) and (**B**) t_b_imp2_ = *f* (%ACN at the start of the gradient, gradient start time—min).

For the given four CMAs, direct modelling was performed. The models with coefficients calculated in terms of coded factors values are given in Table 3. The adequacy of the obtained model was confirmed using an ANOVA test, *R*^2^, adjusted *R*^2^ (adj. *R*^2^), and predicted *R*^2^ (pred. *R*^2^). For t_b_imp3,_ a linear model (Equation (1)) was suggested as suitable, while for responses t_e_bup_, t_b_imp2,_ and t_e_imp2,_ quadratic models (Equation (2)) were proposed. Representative 3D graphs for t_e_bup_ and t_b_imp2_ are given in Figure 2.

y = b_0_ + b_1_A + b_2_B + b_3_C (1)

y = b_0_+ b_1_A + b_2_B + b_3_C + b_12_AB + b_13_AC + b_23_BC + b_11_A^2^ + b_2_B^2^ + b_33_C^2^ (2)

The adequacy of the obtained models was confirmed through *R*^2^, adj. *R*^2^, and pred. *R*^2^ values (Table 3). Regarding the coefficients of the models listed above, they denote the magnitude of the factor’s influence in each response. In addition, the sign implies a response increase by increasing one factor’s values, when its coefficient holds a positive sign. As can be seen from Table 3, the positive sign of b_B_ (coefficient of B factor) implies that an increase in the chaotropic salt’s concentration in the mobile phase evokes an increase in retention time of basic drugs, a piece of evidence consistent with the current knowledge of the retention mechanism of basic analytes with chaotropic salts [28].

In order to achieve the ATP of our study, the separation criteria s_1_ and s_2_ were calculated and then were indirectly modelled:

s_1_ = t_bimp2_ − t_e_bup_ (3)

s_2_ = t_b_imp3_ − t_e_imp2_ (4)

**Figure 3:**
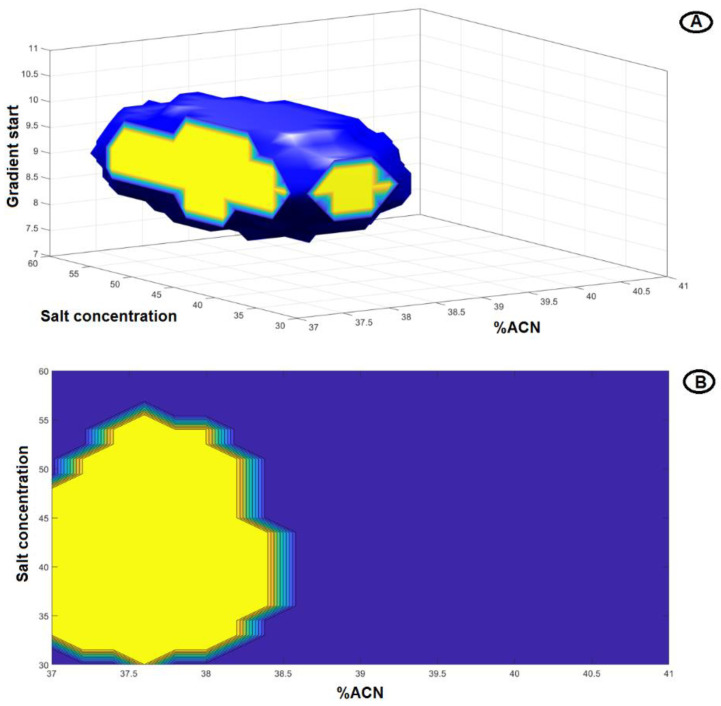
(**A**) 3D representation of DS for the predefined CMAs achieved with probability π ≥ 85%. (**B**) 2D representation of DS after setting a fixed value for C (gradient start time) = 10.0 min. The yellow part corresponds to the region of the design space where the working point should be situated.

Regarding the method performance characteristics, acceptance limits for CMAs (s_1_ ≥ 0, s_2_ ≥ 0) with the desired quality level (π ≥ 85%) were set. Firstly, the knowledge space was gridded by discretization of the numerical parameters giving 21 levels for A × 21 levels for B × 11 levels for C = 4851 combinations of the investigated CMPs. Secondly, Monte Carlo simulations were used to compute the predictive probability for a given CMA to be greater than the desired threshold. Monte Carlo simulations with 5000 iterations were applied to propagate the errors originating from the model coefficients’ calculation and obtain predictive distribution of the CMAs in each of the 4851 points. Finally, the region of the experimental domain fulfilling the aforementioned conditions was computed (Figure 3).

**Figure 4:**
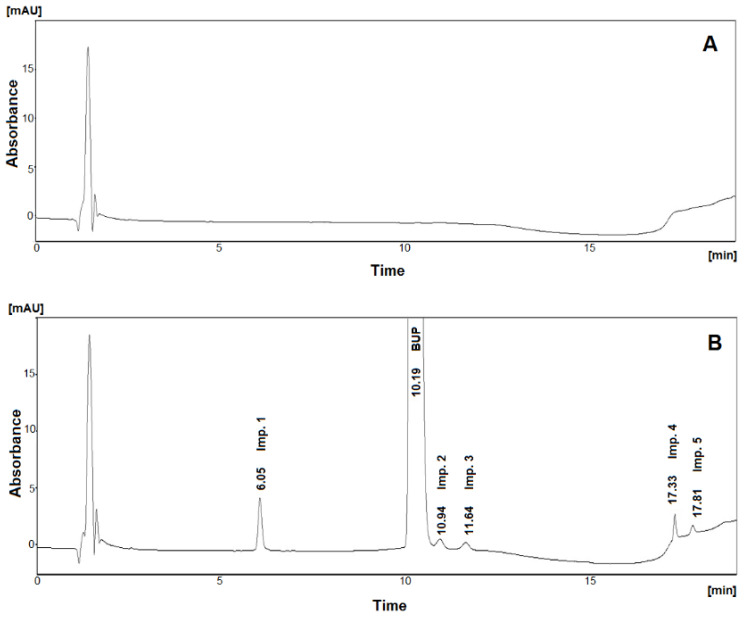
Chromatograms obtained under conditions suggested by the working point: (**A**) placebo mixture of excipients, (**B**) sample solution containing 400 μg/mL BUP spiked with all impurities at their SL.

From the defined DS, as illustrated in Figure 3A, any point can be selected as a working point, i.e., normal operating conditions. In this study, we singled out the following conditions: 37.5% acetonitrile at the start of the linear gradient step (this step lasts for 7 min and at the end, the % ratio of acetonitrile reaches 70%), 10.0 min as the linear gradient step start time, and 45 mM as the concentration of the chaotropic salt. As is illustrated in the 3D visualization of the defined DS (Figure 3A), working points in the middle of the DS were selected for two CMPs, (A) and (B). For CMP (C), a more distinct value from the middle was selected since it exhibited slightly better chromatographic results. The 2D graph for DS, setting the linear gradient step start time equal to 10.0 min, is depicted in Figure 3B. The rest of the normal operating conditions are presented in the Materials and Methods. Relevant chromatograms are presented in Figure 4.

### 2.3. Method Validation

The gradient program, within normal operating conditions, was set by robust optimization and implied 37.5% acetonitrile during the isocratic step of the gradient program from 0 min to 10.0 min; an increase in % ratio of acetonitrile from 37.5% to 70% within the linear gradient step from 10.01 min to 17.0 min; turning back to 37.5% acetonitrile from 17.01 min to 21.0 min; and re-equilibration until 23.0 min. Finally, a validation protocol was performed for the proposed method, based on the international guidelines.

The selectivity study was carried out by comparing chromatograms of the placebo (Figure 4A) and a sample solution containing 400 mg/mL of bupropion hydrochloride spiked with a known amount of all impurities in concentrations according to their specification limits (Figure 4B). No matrix interference was observed.

Method linearity was demonstrated by analyzing five different concentrations of BUP at the concentration range 200–600 μg/mL and each impurity at a concentration span from LOQ to 120% of the specification limit, as depicted in Table 4. The calibration curve for each analyte was calculated using regression analysis and the regression parameters are presented in Table 4, establishing an excellent linearity.

LOD and LOQ were evaluated based on the expressions LOD = 3.3 σ/S and LOQ = 10 σ/S, where σ is the standard deviation of the y-intercepts of the regression line and S is the slope of the calibration curve of each analyte. The obtained values (Table 4) were then confirmed by preparing the samples with analytes at the LOQ and LOD level.

The accuracy of the method was evaluated with recovery experiments of laboratory mixtures and the recovery values found (Table 4) within the acceptance criteria (recovery values: 98.0–102% for the active ingredient, 90.0–110.0% for impurities with a specification limit ≥ 1.0% (imp. 4), 80.0–120.0% for impurities with 0.5% ≤ specification limit < 1.0% (imp. 1) and 70.0–130.0% for impurities with 0.1%≤ specification limit < 0.5% (imp. 2, 3, and 5)) [28].

The precision of the method was assessed by performing three injections at three concentrations for each impurity covering the range from LOQ to 120% of the specification limit and six sample solutions, extracted from tablets, containing 400 μg/mL of BUP. The % relative standard deviations (% RSD) for each were then calculated and are presented in Table 4. The requirements of %RSD (%RSD 2.0% for the active ingredient, 5.0% for impurities with a specification limit ≥ 1.0% (imp. 4), 10.0% for impurities with 0.5% ≤ specification limit < 1.0% (imp. 1) and 15% for impurities with 0.1%≤ specification limit < 0.5% (imp. 2, 3, and 5)) were fulfilled for all analytes [29].

## 3. Materials and Methods

### 3.1. Reagents and Solvents

Reference standards of bupropion hydrochloride and its four impurities (Deschloro Bupropion Hydrochloride—impurity 1, 3-Deschloro-4-chloro Bupropion Hydrochloride—impurity 2, 3-Deschloro-3-bromo Bupropion Hydrochloride—impurity 3 and 1-(3-Chlorophnenyl)-1-hydroxy-2-propanone—impurity 4) were obtained from Toronto Research Chemicals (Toronto, ON, Canada). The fifth impurity (3 Chloropropiophenone—impurity 5) and the chaotropic salt potassium hexafluorophosphate were purchased from Fluorochem (Glossop, UK). Acetonitrile and methanol, both HPLC-grade, and hydrochloric acid were obtained from Merck KGaA (Darmstadt, Germany). Water (HPLC-grade) filtered through Millipore Simplicity (Billerica, MA, USA) was used for preparation of the mobile phase and solutions. All reagents were of analytical grade. Nylon syringe filters were purchased from Sun SRI (Rockwood, TN, USA). Wellbutrin^®^ tablets containing 150 mg of bupropion hydrochloride (GlaxoSmithKline, Athens, Greece) were purchased from a local drugstore and Ph. Eur. quality excipients were used for the preparation of the placebo mixture.

### 3.2. Instrumentation and Chromatographic Conditions

The experiments for method development and validation were carried out on the chromatographic system VWR Hitachi Chromaster (Tokyo, Japan) consisting of an HPLC pump with an on-line degasser, a column oven, an autosampler and a photo-diode array detector, controlled by the Clarity VA v.15.9.0 chromatographic software package from DataApex (Prague, Czech Republic). The partial loop injection volume was 5 μL. Chromatographic separations were performed on a Restek ROC C-18 with 150 mm × 4.6 mm, 3 μm particle size column (Bellefonte, PA, USA) with UV detection at 250 nm. The flow rate was 1 mL/min and the column temperature was set at 35 °C. Mobile-phase compositions at the beginning and at the end of the gradient program, as well as the gradient run times, were varied according to the plan of experiments defined by Box–Behnken design (BBD).

### 3.3. Solutions for Experimental Design

In order to prepare stock solutions, the respective amounts of BUP and impurities were dissolved in methanol. The concentrations of 2 mg/mL for BUP and 100 μg/mL for all the impurities were obtained. Stock solutions were further diluted with methanol–water (50:50, *v*/*v*) to obtain working solutions containing 10 μg/mL of each impurity and 100 μg/mL of BUP.

### 3.4. Solutions for Selectivity Estimation

A placebo mixture of the excipients was prepared in the concentration ratio corresponding to the content in the tablets. It was treated in the same manner as the sample used for precision estimation. A sample solution mixture containing 400 μg/mL bupropion hydrochloride and the impurities at the concentrations corresponding to their specification limits were used to prove the method’s selectivity.

### 3.5. Solutions for the Estimation of Linearity

Five solutions containing BUP were prepared over a concentration range of 200–400 μg/mL and five solutions containing the five impurities over a concentration range of 0.4–2.4 μg/mL for impurity 1, 0.2–1.0 μg/mL for impurities 2 and 3, 1.8–12 μg/mL for impurity 4, and 0.2–0.8 μg/mL for impurity 5.

### 3.6. Solutions for the Estimation of Accuracy

The accuracy was assessed by spiking the placebo mixture with known amounts of the BUP reference standard and its 5 impurities. Regarding BUP, three test solutions were prepared, containing 320, 400, and 480 μg/mL, corresponding to 80, 100, and 120% concentration levels, respectively. Furthermore, the placebo mixture was spiked with the impurities’ reference standards in order to obtain the following concentrations: limit of quantification (LOQ), specification limit (SL), and 120% of the specification limit. The analysis was performed in triplicate.

### 3.7. Solutions for the Estimation of Precision

A certain quantity of pulverized tablets corresponding to 150 mg BUP was placed into a 100 mL volumetric flask. BUP was dissolved in methanol with the assistance of an ultrasonic bath for 30 min. Then, the volumetric flask was placed on the magnetic stirrer for 1 h. The volumetric flask was filled to the mark with the same solvent, and a portion was passed through a nylon filter (0.45 μm pore size). From the filtered solution, a stock solution was prepared containing 1.5 mg/mL BUP. From that stock solution, six solutions containing 400 μg/mL of BUP (SL) were prepared in methanol–water (50:50, *v*/*v*). Regarding the five impurities, the precision was estimated from the replicates of the laboratory mixture prepared for the accuracy testing, as their levels in tablets were below the LOQ value.

### 3.8. Analysis of BUP Tablets

The analysis of Wellbutrin^®^ tablets containing 150 mg of BUP was performed via the current HPLC method. Ten tablets were pulverized; a quantity of tablet content corresponding to 150 mg of BUP was placed into a 100 mL volumetric flask and treated as previously described.

### 3.9. Software

The experimental plan and data analysis were performed using Design-Expert^®^ 13 trial version (Stat-Ease Inc., Minneapolis, MN, USA). Indirect modeling, Monte Carlo simulations, and the graphical presentation of DS were processed in MATLAB^®^ R2018b (MathWorks, Natick, MA, USA).

## 4. Conclusions

In this study, a reliable chaotropic chromatography method for the efficient baseline separation and accurate determination of bupropion and its five impurities in tablets was developed, based on AQbD principles. Potassium hexafluorophosphate salt, exhibiting the highest chaotropic effect among other chaotropic salts, was utilized as a mobile-phase additive, providing better peak symmetry and improved selectivity of basic analytes. Moreover, a 2^6-2^ fractional factorial design and a Box–Behnken design were employed so as to distinguish and optimize the selected CMPs, respectively. The CMAs identified in this study were: a separation criterion between BUP and imp. 2 (s_1_ ≥ 0) and a separation criterion between imp. 2 and imp. 3 (s_2_ ≥ 0). Other than the DoE methodology, Monte Carlo simulations were applied so as to define the DS with the desired quality level (*π* ≥ 85%) and the predefined acceptance limits. The gradient program, within normal operating conditions, was selected from the defined DS setting the method’s final chromatographic conditions. Finally, the method was tested for selectivity, sensitivity, linearity, accuracy, and precision, proving its reliability and applicability in routine pharmaceutical analysis.

## Figures and Tables

**Table 1:** Plan of experiments defined by 2^6-2^ fractional factorial design and the obtained responses.

Runs	Factors						Responses				
	A	B	C	D	E	F	t_e_bup_	t_b_imp2_	t_e_imp2_	t_b_imp3_	t_R_imp5_
1	2.4	37	80	30	8	13	12.53	12.45	12.66	12.71	15.51
2	2.4	43	20	40	8	9	5.89	5.31	5.62	5.55	15.20
3	3.2	40	50	35	6.5	11	9.05	9.03	9.41	9.51	13.29
4	2.4	37	20	40	5	13	8.71	8.67	8.95	9.01	14.07
5	4.0	37	80	30	5	13	10.99	10.89	11.11	11.13	14.27
6	2.4	43	80	40	5	13	6.78	6.71	7.00	7.05	12.38
7	2.4	43	20	30	8	13	5.41	5.32	5.56	5.56	13.84
8	4.0	37	20	40	8	13	9.80	9.83	10.23	10.63	15.24
9	4.0	37	80	40	5	9	9.71	9.55	9.75	9.81	31.50
10	3.2	40	50	35	6.5	11	9.07	9.05	9.34	9.52	13.32
11	2.4	37	80	40	8	9	11.18	10.68	12.52	10.55	33.00
12	2.4	37	20	30	5	9	9.28	9.11	9.29	10.49	32.00
13	2.4	43	80	30	5	9	7.71	7.87	7.97	8.09	32.50
14	4.0	43	20	30	5	13	6.88	6.77	7.01	7.11	12.99
15	4.0	43	20	40	5	9	6.03	5.85	6.11	6.14	11.29
16	3.2	40	50	35	6.5	11	9.01	8.99	9.36	9.51	13.31
17	4.0	37	20	30	8	9	11.11	10.98	11.17	11.17	31.00
18	4.0	43	80	40	8	13	7.10	6.93	7.20	7.25	13.67
19	4.0	43	80	30	8	9	7.86	7.86	8.13	8.32	24.36

**A**, pH of the aqueous part of the mobile phase (pH was adjusted with HCl); **B**, ratio of acetonitrile at the start of the linear gradient step; **C**, concentration of chaotropic agent; **D**, column temperature; **E**, linear gradient step start time; **F**, linear gradient end time; **t_e_bup_**, retention time of BUP peak end; **t_b_imp2_**, retention time of imp. 2 peak beginning; **t_e_imp2_**, retention time of imp. 2 peak end; **t_b_imp3_**, retention time of imp. 3 peak beginning; **t_R_imp5_**, retention time of imp. 5.

**Table 2:** Plan of experiments proposed from software for BBD and the obtained responses.

Runs	Factors			Responses			
	A	B	C	t_e_bup_	t_b_imp2_	t_e_imp2_	t_b_imp3_
1	39.00	45.00	9.00	9.35	9.39	9.65	9.93
2	41.00	45.00	7.00	7.93	7.93	7.93	8.00
3	39.00	60.00	11.00	9.79	9.83	10.13	10.41
4	41.00	45.00	11.00	8.05	8.05	8.05	8.15
5	37.00	30.00	9.00	10.43	10.56	10.85	11.12
6	41.00	30.00	9.00	7.19	7.19	7.47	7.56
7	39.00	60.00	7.00	9.75	9.83	10.03	10.31
8	37.00	45.00	11.00	11.34	11.43	11.81	12.23
9	37.00	45.00	7.00	10.98	11.01	11.21	11.39
10	39.00	45.00	9.00	9.33	9.38	9.65	9.95
11	37.00	60.00	9.00	11.90	12.02	12.37	12.59
12	39.00	45.00	9.00	9.35	9.41	9.61	9.96
13	39.00	45.00	9.00	9.35	9.41	9.68	9.99
14	39.00	30.00	7.00	8.63	8.68	8.93	9.14
15	41.00	60.00	9.00	8.11	8.11	8.39	8.53
16	39.00	30.00	11.00	8.62	8.67	8.92	9.19

**A**, ratio of acetonitrile at the start of the linear gradient step; **B**, concentration of chaotropic agent; **C**, linear gradient step start time; **t_e_bup_**, retention time of BUP peak end; **t_b_imp2_**, retention time of imp. 2 peak beginning; **t_e_imp2_**, retention time of imp. 2 peak end; **t_b_imp3_**, retention time of imp. 3 peak beginning.

**Table 3:** Coefficients of the obtained models and their statistical evaluation.

	t_e_bup_	t_b_imp2_	t_e_imp2_	t_b_imp3_
**b_o_**	9.35	9.4	9.65	9.90
**b_A_**	−1.67 ***	−1.72 ***	−1.80 ***	−1.89 ***
**b_B_**	0.59 ***	0.59 ***	0.59 ***	0.61 ***
**b_C_**	0.063 ***	0.065	0.099 *	0.14 *
**b_AB_**	−0.14	−0.14	−0.15 *	−
**b_AC_**	−0.061	−0.076	−0.12	−
**b_BC_**	0.012	0.00325	0.027	−
**b_A_^2^**	0.22 **	0.21 *	0.19 *	−
**b_B_^2^**	0.16 *	−0.14	−0.063	−
**b_C_^2^**	0.00075	−0.00425	−0.081	−
***R* ^2^**	0.99677	0.9955	0.9977	0.9880
**adj. *R*^2^**	0.9918	0.9888	0.9942	0.9851
**pred. *R*^2^**	0.9476	0.9288	0.9644	0.9751

* Statistically significant coefficients for *p* < 0.05; ** Statistically significant coefficients for *p* < 0.01; *** Statistically significant coefficients for *p* < 0.001. **t_e_bup_**, retention time of BUP peak end; **t_b_imp2_**, retention time of imp. 2 peak beginning; **t_e_imp2_**, retention time of imp. 2 peak end; **t_b_imp3_**, retention time of imp. 3 peak beginning; b_0_ is the intercept. b_A_, b_B_, and b_C_ are the coefficients of main effect terms A, B, and C, respectively; b_AB_, b_AC_, and b_BC_ are coefficients of the interaction terms. b_A_^2^, b_B_^2^, and b_C_^2^ are coefficients of quadratic terms.

**Table 4:** Validation parameters: limit of detection, linearity, and accuracy of the proposed HPLC method.

Compound	LOD (μg/mL)	Linearity				Accuracy (Precision)	
		Concentration Range (μg/mL)	a	b	r	Concentration Level (μg/mL)	% Recovery (% RSD) *
BUP	-	200–400	12.318	−43.145	0.9991	320 (80%) 400 (100%) 480 (120%)	99.7 99.7 (0.17) 99.4
Imp. 1	0.07	0.4–2.4	15.418	0.0397	0.9999	0.4 (LOQ) 2.0 (100%) 2.4 (120%)	99.4 (1.7) 104.1 (0.3) 98.9 (1.8)
Imp. 2	0.06	0.2–1.0	11.558	−0.576	0.9995	0.2 (LOQ) 0.8 (100%) 1.0 (120%)	99.4 (6.2) 104.1 (1.3) 98.9 (0.9)
Imp. 3	0.04	0.2–1.0	9.291	−0.472	0.9998	0.2 (LOQ) 0.8 (100%) 1.0 (120%)	90.3 (3.6) 101 (1.5) 104.5 (1.3)
Imp. 4	0.6	1.8–12	0.847	−0.458	0.9967	1.8 (LOQ) 9.0 (100%) 12 (120%)	99.7 (2.4) 102.1 (1.5) 99.3 (4.6)
Imp. 5	0.06	0.2–0.8	10.511	−0.0792	0.9993	0.2 (LOQ) 0.4 (100%) 0.8 (120%)	102.8 (2.9) 103.4 (0.1) 103.3 (2.4)

**a**, slope; **b**, intercept; **r**, correlation coefficient (acceptance value > 0.99 for active ingredients, >0.98 for related compounds). * %RSD values for the respective concentration levels in parentheses.

## Data Availability

Data is contained within the article.
